# Psychometric properties of a clinical assessment tool in the postgraduate midwifery programme, Botswana

**DOI:** 10.4102/curationis.v46i1.2404

**Published:** 2023-04-04

**Authors:** Itumeleng Rasetshwane, Nombulelo V. Sepeng, Ramadimetja S. Mooa

**Affiliations:** 1 Department of Nursing, Faculty of Health Sciences, University of Pretoria, Pretoria, South Africa

**Keywords:** internal consistency reliability, clinical assessment tool, clinical assessment, psychometric properties, content validity

## Abstract

**Background:**

The psychometric properties of a clinical assessment tool used in the postgraduate midwifery programme in Botswana have not been evaluated. A lack of reliable and valid clinical assessment tools contributes to inconsistencies in clinical assessment in midwifery programmes.

**Objectives:**

This study aimed to evaluate the internal consistency and content validity of a clinical assessment tool used in the postgraduate midwifery programme in Botswana.

**Method:**

For internal consistency, we calculated the total-item correlation and Cronbach’s alpha coefficient. For content validity, subject matter experts completed a checklist to evaluate the relevance and clarity of each competency in the clinical assessment tool. The checklist included questions with Likert-scale responses, indicating the level of agreement.

**Results:**

The clinical assessment tool had a good reliability, with a Cronbach’s alpha of 0.837. The corrected item total correlation values ranged from –0.043 to 0.880 and the Cronbach’s alpha (if item deleted) ranged from 0.079 to 0.865. Overall content validity ratio was 0.95, and content validity index was 0.97. Item content validity indices ranged from 0.8 to 1.0. The overall scale content validity index was 0.97 and the scale content validity index using universal agreement was 0.75.

**Conclusion:**

The clinical assessment tool used in the postgraduate midwifery programme in Botswana has acceptable reliability. Most of the competencies included in the clinical assessment tool were relevant and clear. Certain competencies need to be reviewed to improve the reliability and validity of the clinical assessment tool.

**Contribution:**

The clinical assessment tool currently used in the postgraduate midwifery programme in Botswana had acceptable internal consistency reliability and validity.

## Introduction

Clinical assessment in midwifery training is required to evaluate the students’ actual performance (Embo et al. 2017). Clinical assessment tools are often used to collect evidence of the midwifery students’ clinical performance (Franklin & Melville 2015; Sweet et al. 2017). The psychometric evaluation of these tools helps in the reduction of measurement error and improvement of authentic assessments (Vitoratou & Pickles 2017) that will produce accurate findings or results (Souza, Alexandre & Guirardello 2017). Psychometric properties mainly refer to the reliability and validity of the instrument or clinical assessment tool (Echevarría-Guanilo, Gonçalves & Romanoski 2017). Reliability explains the degree at which the items in a tool or instrument consistently measure the intended attribute (Souza et al. 2017). Reliability can be estimated using three major forms; test–retest reliability, alternate forms of reliability, and internal consistency (Bolarinwa 2015; Hajjar 2018). The evaluation of reliability in this study, will be focused on internal consistency. This is so because the internal consistency measures the internal reliability of the items of a tool or instrument, and gives information on the extent to which these items measure the same thing (Bolarinwa 2015; Souza et al. 2017). Cronbach’s alpha coefficient is commonly recommended and used to measure the internal consistency mainly for instruments or tools that adopt the Likert scales with ascending or descending order of values or categories (Echevarría-Guanilo et al. 2017; Namdeo & Rout 2016).

By assessing reliability, we can estimate the impact of variation on the assessment scores (Mokkink et al. 2019) and determine if the tool is able to assess and measure the midwifery skills and competencies needed to provide quality and holistic midwifery care (Helminen et al. 2016; Löfmark & Mårtensson 2017). However, the reliability of these clinical assessment tools is not often addressed (Morrow et al. 2016) and remains a major challenge in nursing and midwifery (Fisher et al. 2019; Malakooti, Bahadoran & Ehsanpoor 2018; Ossenberg, Dalton & Henderson 2016). As a result, many nursing competency assessment tools were developed but only a few of these were evaluated for reliability (Wu et al. 2016).

A lack of evidence of the evaluation of the psychometric properties of the clinical assessment tools contributes to the challenges and inconsistencies in clinical assessment (Morrow et al. 2016). Consequently, these inconsistencies in clinical assessment may result in assessor bias, different interpretations of the midwifery students’ clinical performance and the variations in the quality of assessment (Helminen et al. 2016; Najjar, Docherty & Miehl 2016). Therefore, evaluation of the psychometric properties of the clinical assessment tools used in midwifery is vital to improve the quality of assessment and to produce accurate results or evidence (Nartgün & Şahin 2015) that are required to make conclusions as to whether the midwifery students have achieved the stipulated clinical competencies.

Apart from reliability, the clinical assessment tools should be valid. Content validity is usually assessed by experts and provides preliminary evidence of the tool’s construct validity (Zamanzadeh et al. 2015). It measures the representativeness and clarity of items and the extent to which they accurately assess the intended construct (Yusoff 2019). Content validity usually gives the preliminary information regarding the (construct) validity of a tool (Almanasreh, Moles & Chen 2019). On the other hand, criterion validity measures the ability of the tool to measure a certain concept or results against another instrument (Bolarinwa 2015). Measuring these forms of validity before determining content validity of a tool, may jeopardise the instrument’s quality (Almanasreh et al. 2019).

Not assessing the reliability and validity of the clinical assessment tools prevents refinement and development of quality assessment tools, which may lead to frustrated educators and students (Vitoratou & Pickles 2017). Navabi et al. (2016) in their development and validation of evaluation tools of nursing students’ clinical pharmacology, found out that 41% of midwifery students were frustrated about the inadequacy of the evaluation processes. Kassab and Hamadneh (2019) reported that there was no reliable tool to evaluate the midwives’ basic new-born resuscitation skills in the delivery rooms in Jordanian healthcare facilities. On the other hand, Blaze and Mariam (2021) reported that there is shortage of psychometrically validated competence assessment tools for midwifery students in Bangalore, India.

Similar problems abound in Botswana, where the psychometric properties, the reliability and validity of the clinical assessment tool have not been evaluated. In Botswana, the midwifery curriculum for postgraduate students runs for four semesters over 2 years. Each semester comprises a theoretical and clinical component. Postgraduate midwifery students are placed and rotate in different midwifery related clinical areas. These students are graded using the same clinical evaluation tool, which was developed by the Midwifery Task Force and adopted in the midwifery postgraduate curriculum in 2011 (Ministry of Health 2011). However, there is no evidence that this tool was evaluated for reliability and validity. In this study, we evaluate the internal consistency and content validity of the clinical assessment tool used in the postgraduate midwifery programme in Botswana. Our research questions include the following:

- What is the internal consistency of the clinical assessment tool used in the post graduate midwifery programme in Botswana?
- What is the content validity of the clinical assessment tool used in the postgraduate midwifery programme in Botswana?

## Research design and methods

### Study design

The study used a methodological research design.

### Setting and participants

The study was conducted in the four midwifery training schools in Botswana, namely the Bamalete Lutheran School of Nursing, the Kanye Seventh Day Adventist College of Nursing, the Institute of Health Sciences-Gaborone, and the Institute of Health Sciences, Francistown. These training schools offer a Diploma in Advanced Midwifery and include the clinical areas in antenatal, family planning, intra-partum, post-partum and neonatal care.

The same clinical assessment tool is used by all the four midwifery training schools to evaluate the students. We randomly selected 10 subject matter experts (SMEs) to determine the content validity of the clinical assessment tool. Eight of the SMEs were in academia and two were from the Nurses and Midwifery Council of Botswana. According to Almanasreh et al. (2019), there is no consensus on a minimum number of experts to determine content validity and often up to 10 experts are used. We included SMEs who had a midwifery specialty at master’s degree level and above, more than 2 years in midwifery teaching, experience of developing clinical assessment tools and being involved in regulating midwifery training and practice.

### Data collection tools and procedures

We calculated the internal consistency from the 114 completed clinical assessment tools for midwifery postgraduate students. After obtaining the required permission, data were collected from the clinical assessment tools and the SMEs. The SMEs were informed about the study’s aims, objectives, expectations, and the legal and ethical implications. The SMEs who agreed to participate provided their e-mail addresses and we then sent individual e-mails to each SME. The e-mail included a participant information letter, a consent form, a confidentiality agreement form, and the data collection tools for relevance and clarity.

The clinical assessment tool used in the postgraduate midwifery programme measures 12 competenciesthat is adequate relevant history, preparation for procedure, prioritises and takes appropriate action, manual dexterity, interpretation of findings, provides relevant education, time management, relates theory to practice, critical thinking, conducts self in a professional, caring and empathetic approach, and reporting. These competencies are rated using a Likert scale from 0 (poor performance) to 5 (high performance).

To determine content validity, the 10 SMEs completed electronic checklists, where they evaluated the relevance and clarity of each competency on the clinical assessment tool. We sent weekly emails to the SMEs, reminding them to complete the checklists, until all the checklists were received. The checklists were specifically developed to evaluate the relevance and the clarity of the content of the clinical assessment tool. We used an approach similar to Bolarinwa (2015) and Zamanzadeh et al. (2015) to evaluate content validity. We asked SMEs to rank the relevance and clarity of each item using a four-point scale for relevance and a three-point scale for clarity (Bolarinwa 2015).

### Data analysis

Data from the assessments’ tools were captured in an excel sheet and exported to Statistical Package for the Social Sciences (SPSS) version 25 for analysis. We estimated the internal consistency using Cronbach’s alpha to represent the homogeneity of the clinical assessment tool. Cronbach’s alpha values were used to estimate the overall reliability of the tool. We also calculated the corrected item total correlation (C-ITC) and Cronbach’s alpha if item deleted to further explore the internal consistency of the clinical assessment tool.

From the SMEs responses, we calculated the content validity ratio (CVR) and content validity index (CVI), specifically looking at the relevance and clarity of each item. The CVR was calculated using the formula:


(ne−N/2)/(N/2) [Eqn 1]

where *ne* is the number of experts who rated the competency as relevant and *N* being the total number of experts (Mobaraki, Ghavami & Gol 2019). Using the Lawshe’s model of 1975, a CVR value of 0.62 or more was deemed acceptable because we had a panel of 10 experts (Tanriöğen & Kurban 2017). The CVI was calculated using the formula: number of experts rating each item 3 as “very clear” in clarity and rating item 3 as “quite relevant” or 4 as “highly relevant” in relevance divided by number of experts (*N*). A CVI value over 0.79 was considered acceptable (Mobaraki et al. 2019). We also calculated item content validity index (I-CVI), average of item content validity on the scale (S-CVI/Ave), and scale level content validity index based on universal agreement (S-CVI/UA) to further explore the content validity of the clinical assessment tool.

### Ethical considerations

The Faculty of Health Sciences Research Ethics Committee at the University of Pretoria approved the study (Ethics reference no., 285/2020). The study was also approved by the Health Research Unit in the Ministry of Health, Botswana (Reference no. HPDME 13/18/1). Permission was further obtained from the Institutional Review Boards (IRBs) and heads of the Midwifery Programme departments where the clinical assessments were completed. Although the study had no foreseeable physical discomfort or risk, the research was guided by the principles of respect for human dignity, beneficence and justice.

## Results

### Characteristics of participants

Most of the participants (*n* = 9) were women. Four SMEs were midwifery lecturers from the University of Botswana, two were midwives from the Botswana Nursing and Midwifery Council and four were midwifery lecturers and administrators from the different nursing and midwifery training institutions in Botswana. The socio-demographic profile of the 10 SMEs is summarised in Table 1.

**TABLE 1:** Socio-demographic profile of the SME (*n* = 10) who assessed the content validity of a clinical assessment tool for postgraduate midwifery students in Botswana.

SME	Gender	Age (years)	Facility/institution	Designation	Years of experience	Highest qualification
1	M	54	University of Botswana	Lecturer	9	PhD Lit. and Philosophy and Post Graduate in Midwifery
2	F	50	University of Botswana	Lecturer (midwifery)	16	Masters in Midwifery
3	F	52	Bamalete of School of Nursing	Principal (midwifery)	29	Masters in Midwifery
4	F	56	Institute of Health Sciences, Gaborone	Head of Department(midwifery)	31	Post Graduate in Midwifery and PhD in Counselling
5	F	50	Nursing and Midwifery Council, Botswana	Registrar (midwife)	34	Masters in Midwifery
6	F	56	Molepolole Institute of Health Sciences	Deputy Principal/midwife	35	Masters in Midwifery
7	F	52	University of Botswana	Lecturer	29	Post Graduate in Midwifery and Masters in Nursing
8	F	51	University of Botswana	Lecturer	20	Masters in Midwifery
9	F	52	Bamalete of School of Nursing	Head of Department (midwifery)	12	Masters in Midwifery
10	F	51	Nursing and Midwifery Council, Botswana	Deputy Registrar (midwife)	30	Masters in Nursing (Women’s Health)

SME, subject matter expert, M, male; F, female.

### Internal consistency

The overall Cronbach’s alpha of the clinical assessment tool was 0.837, indicating good internal consistency (*n* = 114). We also evaluated the internal consistency using C-ITC and Cronbach’s alpha if item deleted (Table 2). The C-ITC of the 12 competencies ranged between −0.043 and 0.880. The clinical assessment tool had seven competencies with low C-ITC < 0.50. Out of the seven, three competencies had C-ITC > 0.30. These were: manual dexterity (0.496), provides relevant education and counselling (0.404), and relates theory to practice (0.496). These indicated acceptable internal consistency and inter-relatedness among each other. The other four competencies had a low C-ITC below < 0.30, which indicated unacceptable internal consistency and poor correlation among each other. These were knowledge of drugs (−0.043), time management (0.000), conducts self in a professional caring empathetic manner (0.000), and reporting and recording (0.000).

**TABLE 2:** Internal consistency by item analysis (*N* = 114 clinical assessments) of the clinical evaluation tool used in the postgraduate midwifery programme in Botswana.

Clinical assessment tool items	Corrected item-total correlation (C-ITC)	Cronbach’s alpha (α) if item deleted
1. Adequate relevant history and significance	0.880	0.790
2. Preparation for procedure: client, equipment, environment	0.643	0.812
3. Manual dexterity	0.496	0.825
4. Prioritises and takes appropriate action with emerging needs	0.880	0.790
5. Knowledge of drugs used	−0.043	0.865
6. Interpretation of findings	0.880	0.790
7. Provides relevant education and counselling (IEC)	0.404	0.845
8. Time management	0.000	0.844
9. Relates theory to practice	0.496	0.825
10. Critical thinking in client’s care	0.880	0.790
11. Conducts self in a professional, caring empathetic approach	0.000	0.844
12. Reporting and recording	0.000	0.844

IEC, information education and counselling.

The clinical assessment tool had five competencies with C-ITC > 0.50, ranging from 0.643 to 0.880. These indicated good internal consistency with the other items in the clinical assessment tool and were assessing the same attribute (Table 2). These are preparation for procedure: client, equipment and environment (C-ITC = 0.6430), adequate relevant history (C-ITC = 0.880), prioritises and takes appropriate action with emerging needs (C-ITC = 0.880), interpretation of findings (C-ITC = 0.880), and critical thinking in the client’s care (C-ITC = 0.880).

Cronbach’s alpha if item deleted ranged from 0.790 to 0.865 for the 12 competencies. Seven competencies had a significant reduction of more than 0.10 in Cronbach’s alpha values if item was deleted, indicating varied internal reliability (Table 2). These items were; adequate relevant history and significance (α = 0.837–0.790), preparation for procedure: Client, equipment, environment (α = 0.812), manual dexterity (α = 0.837–0.825), prioritises and takes appropriate action with emerging needs (α = 0.837–0.790), interpretation of findings (α = 0.837–0.790), relates theory to practice (α = 0.837–0.825), and critical thinking in client’s care (α = 0.837–0.790).

The other five competencies increased the values of Cronbach’s alpha if item deleted, which also indicated the varied internal consistency (Table 2). These are knowledge of drugs used (α = 0.837–0.865), provides relevant education and counselling (α = 0.837–0.845), time management (α = 0.837–0.844), conducts self in a professional, caring empathetic approach (α = 0.837–0.844), and reporting and recording (α = 0.837–0.844).

### Content validity

All the competencies except manual dexterity, knowledge of drugs used, and reporting and recording were rated as relevant by the SMEs, with I-CVI and CVR values = 1, which indicated good content validity (Table 3). Manual dexterity (I-CVI = 0.8, CVR = 0.6), knowledge of drugs used (I-CVI = 0.9, CVR = 0.80) and reporting and recording (I-CVI = 0.9, CVR = 0.8) still had I-CVI and CVR values of greater than 0.5, and still had good content validity in terms of relevance. In terms of clarity, SMEs agreed that most of the items were clear with good content validity (I-CVI values = 0.7 to 0.9) (Table 3). The competency manual dexterity had the lowest I-CVI value = 0.3, indicating that this item was not clear and may require some revision (Table 3). The results of this study indicated the S-CVI/Ave = 0.97 and that the S-CVI/UA = 0.75.

**TABLE 3:** Content validity (*n* = 10, subject matter experts) showing relevance and clarity of the clinical assessment tool used in the postgraduate midwifery programme in Botswana.

Competencies	Relevance				Clarity		
	I-CVI	CVR	Experts Agreeing	Interpretation	I-CVI	Experts agreeing	Interpretation
1. Adequate relevant history with significance	1.0	1.0	10/10	Excellent	0.7	7/10	Excellent
2. Preparation for procedure: client, equipment, environment	1.0	1.0	10/10	Excellent	0.9	9/10	Excellent
3. Manual dexterity	0.8	0.6	8/10	Excellent	0.3	3/10	Needs revision
4. Prioritises and takes appropriate action with emerging needs	1.0	1.0	10/10	Excellent	0.8	8/10	Excellent
5. Knowledge of drugs used	0.9	0.8	9/10	Excellent	0.7	7/10	Excellent
6. Interpretation of findings	1.0	1.0	10/10	Excellent	0.8	8/10	Excellent
7. Provides relevant education and counselling (IEC)	1.0	1.0	10/10	Excellent	0.9	9/10	Excellent
8. Time management	1.0	1.0	10/10	Excellent	0.8	8/10	Excellent
9. Relates theory to practice	1.0	1.0	10/10	Excellent	0.9	9/10	Excellent
10. Critical thinking in client’s care	1.0	1.0	10/10	Excellent	0.8	8/10	Excellent
11. Conducts self in a professional, caring empathetic approach	1.0	1.0	10/10	Excellent	0.7	7/10	Excellent
12. Reporting and recording	0.9	0.8	9/10	Excellent	0.7	7/10	Excellent

Note: S-CVI/average: relevance – 0.97, clarity – SCVI/UA = 0.75.

I-CVI, item content validity index; CVR, content validity ratio; IEC, information education and counselling; S-CVI, scale-level content validity index.

## Discussion

We evaluated the reliability (internal consistency) and content validity of the clinical assessment tool used in the postgraduate midwifery programme in Botswana. The internal consistency was computed using Cronbach’s alpha coefficient and the C-ITC. Content validity was established through the 10 midwifery experts from the academia and professional field.

The clinical assessment tool used in the postgraduate midwifery programme in Botswana had good internal consistency (Cronbach’s α = 0.837). A Cronbach’s alpha value of 0.70 or above is regarded as good reliability (Bolarinwa 2015; Souza et al. 2017). This indicates that the clinical assessment tool is reliable to assess the midwifery students’ competence. The results of this study also showed some similarities with the results of other studies performed. Shokuhi et al. (2020) evaluated the Postpartum Distress Measure Scale, which showed a reliability of α = 0.94, Kassab and Hamadneh (2019) evaluated the reliability of a questionnaire to measure the midwives’ basic skills on new-born resuscitation and reported a reliability of α = 0.851. These results indicated good reliability of the tools.

The items of the clinical assessment tool showed varied stability as indicated by the C-ITC and Cronbach’s alpha if item deleted. Seven items had low to fair C-ITC, which significantly reduced Cronbach’s alpha values, with a range of 0.12–0.47 (Table 2). This implies that the items are not correlating with each other that might imply that they are overly broad (Paulsen & Brckalorenz 2017). Hajjar (2018) and Namdeo and Rout (2016) cautioned that items with a C-ITC value < 0.50 and significantly reduce or increase the values for Cronbach’s alpha if item deleted, by > 0.10, are not internally consistent with the rest of the items, hence, should be removed from the tool or be reviewed. In our study, the items: manual dexterity and relates theory to practice, had moderately low C-ITC (0.496) and had significant reduction (0.12 each) in the Cronbach’s alpha if item deleted. When an item is deleted and the Cronbach’s alpha drops significantly, the internal consistency of the clinical assessment tool is affected (Souza et al. 2017). The ability of the tool to measure what it is intended is reduced. These have not reduced the Cronbach’s alpha too much; thus, the items are shown to contribute positively to the internal consistency of the clinical assessment tool and should be included in the tool even though they had a C-ITC< 0.50 (Hajjar 2018).

However, the competency knowledge of drugs had a very low C-ITC < 0.50 (−0.043) and significantly resulted in higher scores in Cronbach’s alpha if item deleted (> 0.10). The presence of this item affected the stability of the whole clinical assessment tool. The knowledge of drugs item thus needs to be reviewed or excluded to improve the internal consistency of the clinical assessment tool as it might indicate some redundancy in the clinical assessment tool (Echevarría-Guanilo et al. 2017). Tsogbadrakh et al. (2021) used a similar approach to justify the exclusion of three items with low total-item correlation (< 0.30) when developing a Quality Nursing Care scale in Mongolia (QNCS-M). Kassab and Hamadneh (2019) also removed items that increased the Cronbach’s alpha if item deleted by > 0.10.

On the other hand, the three competencies with C-ITC 0.000 (< 0.30) had insignificant increase (0.07 each) in Cronbach’s alpha if item deleted (Table 2). These items seem to be required to remain in the tool. However, they also need to be reviewed to improve their internal consistency, the reliability of the clinical assessment tool as well as the validity of the results in clinical assessment (Paulsen & Brckalorenz 2017). In contrast, the competencies with good C-ITC (> 0.50) dropped the Cronbach’s alpha scores if item deleted too much (Table 2). The competencies also showed poor correlation and internal consistency and that they are not measuring the same construct. These items need to be reviewed to improve their internal consistency, which will enhance the reliability of the tool in clinical assessments. These competencies might indicate some redundancy (Echevarría-Guanilo et al. 2017).

There was a high level of agreement that the assessment tool was relevant and clear. This study found the CVR and CVI values of 0.95 and 0.97 to be satisfactory, indicating excellent content validity. Kassab and Hamadneh (2019) reported a CVR of 0.94 and CVI of 0.712 indicating that their instrument was valid and could be used to evaluate the midwives’ basic new-born resuscitation skills. Moskoei et al. (2017) also found a CVR of 0.88 and CVI of 0.97 in the methodological study they conducted to evaluate the psychometric properties of the rating scale for clinical competency for mental health nurses. The SMEs agreed that the clarity of most competencies was excellent (Table 3), hence good to excellent content validity of the clinical assessment tool. This implies that the competencies are assessing the intended domain. However, the item for manual dexterity was least clear, with low I-CVI (Table 3). This competence was prone to different interpretations by the clinical assessors. This will in turn affect the grading of students and consistency in assessment (Sweet et al. 2017). The item needs to be reviewed to improve its clarity. In their study, Zamanzadeh et al. (2015) eliminated all items with a CVI lower than 0.70 and reviewed items with a CVI value between 0.70 and 0.79, to improve the validity of the patient centred communication instrument.

### Strength and limitations

This was, to the best of our knowledge, the first methodological study to assess the internal consistency and content validity of the clinical assessment tool used in Botswana’s postgraduate midwifery programme. Using the methodological design, we obtained both the reliability of the tool and the content validity from the experts, and this information aided in determining which aspects of the tool need to be revised or removed to improve its reliability and validity.

This study had some limitations as well, because it has evaluated only the internal consistency and content validity, whereas the other forms of reliability and validity can be evaluated/reviewed to further establish the reliability and validity of the tool.

## Conclusion

The clinical assessment tool currently used in the postgraduate midwifery programme in Botswana had acceptable internal consistency reliability and acceptable content validity. Credibility to the assessment process was added and the areas for improving the assessment tool were highlighted. The clinical assessment tool can be used to assess the midwifery students’ clinical competence. The results of this study provide information that can enable the policymakers, especially in the midwifery curriculum designing and reviews, to have clinical assessment tools that accurately evaluate student’s competence. Nevertheless, further validation of the tool’s reliability and validity using other forms/types of reliability and validity, is required.
